# Possible Autochthonous Malaria from Marseille to Minneapolis

**DOI:** 10.3201/eid1308.070143

**Published:** 2007-08

**Authors:** Barbara Doudier, Hervé Bogreau, Aaron DeVries, Nicolas Ponçon, William M Stauffer, Didier Fontenille, Christophe Rogier, Philippe Parola

**Affiliations:** * Hôpital Nord, Marseille, France; † Institut de Médecine Tropicale du Service de Santé des Armées, Marseille, France; ‡ University of Minnesota, Minneapolis, Minnesota, USA; § Institut de Recherche pour le Développement, Montpellier, France

**Keywords:** malaria, autochthonous, France, Minnesota, dispatch

## Abstract

We report 2 cases of *Plasmodium falciparum* malaria in southern France in a French woman and an American man of Togolese origin who reported no recent travel to malaria-endemic countries. Both infections occurred after a stay near Marseille, which raises the possibility of autochthonous transmission. Entomologic and genotypic investigations are described.

Endemic malaria was eradicated from France during the middle of the past century. However, *Plasmodium falciparum* malaria has recently been reported in several Western European countries in persons with no history of travel or blood transfusion (*1*–*3*). The most recent case of an autochthonous case of vivax malaria was reported in 2006 in Corsica, where susceptible vectors persist (*4*). In southern France, a favorite destination for tourists, the last malaria cases in patients without any recent travel in malaria-endemic areas were reported in 1994 and 2000 (*5*,*6*). We report 2 cases that occurred in southern France during early spring and early summer 2006.

## The Cases

On June 30, 2006, a 41-year-old woman was admitted to the North University Hospital in Marseille, France, with a 12-day history of fever, chills, and diarrhea. She had never traveled outside Europe and had no history of blood transfusion or injection drug use. She was born in Marseille and had lived there her entire life. Her home residence was >20 km from the nearest airport and 10 km from the seaport. The case-patient did not work outside the home and did not report any recreational activities near either location. Her neighborhood included families of Comorian descent. Laboratory testing demonstrated low leukocyte and erythrocyte counts and thrombocytopenia. Peripheral blood smear also demonstrated intra-erythrocytic forms consistent with *P. falciparum* infection with a parasitemia level of 0.1%. *P. falciparum* antigen was detected (Test NowICT; Fumouze, Levallois-Perret, France) and subsequently confirmed by PCR. Treatment comprised a 3-day regimen of quinine-clindamycin (*7*) and the case-patient recovered.

A 34-year-old man with tactile fever, bilateral frontal headache, and diarrhea was examined at a clinic in Minneapolis, Minnesota, on May 1, 2006. This man had lived in Togo, his birth country, all of his life until he emigrated to Minnesota in April 2000. Before becoming ill, he had traveled to Paris and from Paris by train to Marseille on April 7, 2006. Also during this timeframe, he reported a day trip to Camargue (Sorgues, France) where he sustained multiple mosquito bites. On April 24, 2006, he returned to Paris by train and then traveled by air a few hours later to Minneapolis. The first symptoms of malaria developed on April 27. His medical history included 4 episodes of malaria in childhood (none in the past decade) and no blood transfusions. On his last visit to Togo in December 2004, the case-patient had reported no chemoprophylaxis for malaria and had experienced no illness or intervening fevers since that time. Physical examination showed a moderately ill person with hematologic test results indicating thrombocytopenia and intra-erythrocytic ring forms consistent with *P. falciparum* malaria with a 3% parasitemia level. PCR testing confirmed *P. falciparum.* He was given 4 tablets of atovoquone-proguanil once a day for 3 days and recovered completely.

We conducted entomologic surveillance on July 26, 2006, in and around the first case-patient’s residence in Marseille, 26 days after she became ill. No adult mosquitoes were found in the patient’s home, on external staircases, in cellars located around the patient’s home, or in surrounding buildings. Two pools of standing water were identified: a single pool (1 m^2^, 2 cm deep) within a cellar in her building and another pool (10 m^2^, 5 cm deep) located 20 m outside the building. No other containers or places that could collect water were identified. Several *Culex theileri* larvae were identified in the outdoor pool, but there was no evidence of *Anopheles* larvae.

DNA was extracted from blood samples by using the ENZA blood DNA kit according to the manufacturer’s recommendations (Biofidal, Vaulx en Velin, France) and eluted in 100 µL of elution buffer per 250 µL of whole blood. Microsatellites loci (C4M79, Pf2689, TRAP, Pf2802, 7A11, and C4M69) were genotyped by fluorescent end-labeled PCR. Primers sequence, PCR conditions, and methods for genotyping have been described elsewhere (*8*,*9*). Drug-resistant mutations were genotyped. Genotyping of the 2 isolates demonstrated different alleles for the 6 microsatellite loci. We also observed different alleles from codon 59 (dihydrofolate reductase), 437 (dihydropteroate synthase), and 76 (*P. falciparum* chloroquine-resistance transporter) (Tables 1, 2).

**Table 1:** Microsatellite loci genotyping and single nucleotide polymorphism in drug-resistant genes

	C4M79	Pf2689	Pf2802	7A11	C4M69	TRAP
Autochthonous 1*	190	87	141	88	378	132
Autochthonous 2†	221	95	138	99	364	149
3D7‡	221	87	138	94	364	137
W2‡	188	87	146	109	319	140

*Patient 1, *Plasmodium falciparum* diagnosed in France. †Patient 2, *P. falciparum* diagnosed in the United States. ‡Two parasite strains (3D7 and W2) were genotyped as a positive control; water was used as a negative control.

**Table 2:** Two strains of *Plasmodium falciparum* obtained from 2 patients with no recent travel history in malaria-endemic areas*

	Dhfr						Dhps						Pfcrt
	51	16	108	164	59		613	540	581	436	437		76
Autochthonous 1†	–	GCA	AAC	ATA	TGT		GCC	AAA	GCG	TCT	GCT		ACA
Autochthonous 2‡	ATT	GCA	AAC	ATA	CGT		GCC	AAA	GCG	TCT	GGT		AAA
3D7§	AAT	GCA	AGC	ATA	TGT		GCC	AAA	GCG	TCT	GGT		AAA
W2§	ATT	GCA	AAC	ATA	CGT		TCC	AAA	GCG	TTT	GGT		ACA

*Dhfr, dihydrofolate reductase gene; Dhps, dihydropteroate synthase; Pfcrt, *P. falciparum* chloroquine-resistance transporter. Underlines indicate positions that can be mutated. †Patient 1, *P. falciparum* diagnosed in France. ‡Patient 2, *P. falciparum* diagnosed in the United States. §Two parasite strains (3D7 and W2) were genotyped as a positive control; water was used as a negative control.

## Conclusions

The World Health Organization defines introduced autochthonous malaria as that acquired by mosquito transmission from an imported case in an area where malaria is not a regular occurrence (*10*) In France, it is rare to find *P. falciparum* in a blood smear from patients who have not traveled to a malaria-endemic area in the previous 12 months. History of transfusion, organ transplantation, intravenous drug use, or mother-to-fetus transmission must also be excluded (*10*).

In Europe, some recent autochthonous malaria cases have been related to close proximity to airports and shipping ports receiving flights and water craft from malaria-endemic areas (*5*,*6*,*11*). Inadvertent carriage of infective *Anopheles* mosquitoes by airplane, ship, baggage, or bilge water may be responsible for these occurrences. Also, large populations of migrants from areas highly endemic for malaria (*12*) may act as human reservoirs for potential gametocyte carriers. Marseille has a large population of persons of Comorian origin, and most of the patients with imported malaria cases diagnosed in Marseille contracted the disease during a trip to the Comoros Islands (*13*,*14*). Our first case-patient’s malaria may have been linked to her neighborhood, which included Comorian families who had recently traveled to Comoros. Despite the absence of *Anopheles* larvae near the first patient’s neighborhood, local transmission cannot be excluded during the late spring and summer. Summer temperatures in Marseille induce a short *P. falciparum* sporogonic cycle (≈11 days at 28°C), which is compatible with mosquitoes’ longevity at that time.

The Minnesota case-patient likely represents a second case of autochthonous malaria. First, this patient had no illness to suggest an untreated, active infection since his last trip to Togo. Second, even if he had been persistently infected with *P. falciparum* and was semi-immune, this case would still represent an extremely long incubation period (16 months). Third, onset of symptoms was acute, hematologic testing showed infection with a 3% parasitemia level, and the case-patient was moderately ill, which suggests a more recent exposure. Moreover, the genotype was monoallelic for each locus, indicating a clonal infection, which would be expected in areas of relatively low transmission frequency. In contrast, in areas of frequent endemic transmission, such as Togo, multiple allelic polymorphisms would be expected (*9*). Finally, genotyping demonstrated different clones of *P. falciparum*; this finding suggests potential multiple introductions, temporally related, of the parasite into the environment

The last malaria focus in continental France occurred near the end of World War II in the Camargue region, which was visited by our second case-patient in April 2006. However, large *Anopheles* populations, including potential vectors such as *An. hyrcanus*, *An. melanoon*, and *An. atroparus,* are still present from March to November (N. Ponçon and D. Fontenille, unpub.data) in southeastern France, generating an “anophelism without malaria” situation (*15*).

Migrants from malaria-endemic countries, climate, and *Anopheles* populations make southern France a favorable area for sporadic cases of autochthonous malaria in Europe. Given these 2 temporally related cases, clinicians should suspect malaria in patients with unexplained fevers who have recently traveled to areas of southern France.
